# Fault Diagnosis for Rotating Machinery: A Method based on Image Processing

**DOI:** 10.1371/journal.pone.0164111

**Published:** 2016-10-06

**Authors:** Chen Lu, Yang Wang, Minvydas Ragulskis, Yujie Cheng

**Affiliations:** 1 School of Reliability and Systems Engineering, Beihang University, Xueyuan Road No.37, Haidian District, Beijing, China; 2 Science & Technology on Reliability and Environmental Engineering Laboratory, Xueyuan Road No.37, Haidian District, Beijing, China; 3 Research Group for Mathematical and Numerical Analysis of Dynamical Systems, Kaunas University of Technology, Studentu 50-146, Kaunas LT-51368, Lithuania; Jiangnan University, CHINA

## Abstract

Rotating machinery is one of the most typical types of mechanical equipment and plays a significant role in industrial applications. Condition monitoring and fault diagnosis of rotating machinery has gained wide attention for its significance in preventing catastrophic accident and guaranteeing sufficient maintenance. With the development of science and technology, fault diagnosis methods based on multi-disciplines are becoming the focus in the field of fault diagnosis of rotating machinery. This paper presents a multi-discipline method based on image-processing for fault diagnosis of rotating machinery. Different from traditional analysis method in one-dimensional space, this study employs computing method in the field of image processing to realize automatic feature extraction and fault diagnosis in a two-dimensional space. The proposed method mainly includes the following steps. First, the vibration signal is transformed into a bi-spectrum contour map utilizing bi-spectrum technology, which provides a basis for the following image-based feature extraction. Then, an emerging approach in the field of image processing for feature extraction, speeded-up robust features, is employed to automatically exact fault features from the transformed bi-spectrum contour map and finally form a high-dimensional feature vector. To reduce the dimensionality of the feature vector, thus highlighting main fault features and reducing subsequent computing resources, t-Distributed Stochastic Neighbor Embedding is adopt to reduce the dimensionality of the feature vector. At last, probabilistic neural network is introduced for fault identification. Two typical rotating machinery, axial piston hydraulic pump and self-priming centrifugal pumps, are selected to demonstrate the effectiveness of the proposed method. Results show that the proposed method based on image-processing achieves a high accuracy, thus providing a highly effective means to fault diagnosis for rotating machinery.

## Introduction

Rotating machinery, an important and common component in most critical systems, is vital to the reliable operation of the entire system. An unexpected failure of a rotating machinery may cause sudden breakdown of the whole machinery equipment, bringing about enormous financial losses or even personnel casualties [1, 2]. Therefore, condition monitoring and fault diagnosis of rotating machinery is of utmost significance for security and reliability in industrial manufacturing [3, 4].

Rotating machinery under an abnormal state is accompanied with changes in vibration [5]. Thus, the vibration signal analysis method has been widely applied to fault diagnostics of rotating machinery. Feature extraction is a vital stage that determines diagnosis accuracy [6]. Considerable research has been performed on feature extraction based on signal decomposition[7], among which self-adaptive decomposition methods have been widely spread. Empirical mode decomposition (EMD) [7–10] and local mean decomposition (LMD) [11, 12] are two typical self-adaptive decomposition methods. However, problems such as envelope error, mode mixing and end effect exist in EMD and LMD, which directly influence the diagnostic accuracy [12, 13]. Furthermore, EMD and LMD are usually used combined with time domain feature extraction, such as complexity measures [14], skewness, and kurtosis [15]. The selection of the time domain features is usually performed manually by field experts, whose knowledge and skill make difference in the diagnosis accuracy. The above disadvantages limit the application of these methods in practice. Therefore, it is very necessary to put forward a fault diagnosis method which can realize automatic feature extraction with a high accuracy and strong robustness.

Nowadays, with the rapid development of science and technology, fault diagnosis methods based on multi-disciplines have attracted more and more attention of researchers in the field of fault diagnosis. Many interdisciplinary methods have been proposed for fault diagnosis. In reference [16], genetic algorithm (natural evolution theory) combined with support vector machine (machine learning) is applied to bearings for fault diagnosis. In reference [17], ant colony algorithm (bionics) is employed to realize fault diagnosis for rotating machinery. However, few studies have been reported to realize fault diagnosis for rotating machinery employing calculation methods in the field of image processing. Essentially, fault diagnosis for rotating machinery is a process of fault mode recognition, which is extremely similar to the process of image recognition. Both fault classification and image recognition belong to the category of pattern recognition. Therefore, it is of high feasibility to introduce the calculation methods in the field of image processing to fault diagnosis for rotating machinery.

In this paper, a novel approach based on image processing is presented to realize automatic feature extraction for rotating machinery with a high accuracy and strong robustness and finally realize fault classification.

To introduce the computing method of image processing to the field of fault diagnosis, first it is necessary to convert the vibration signal into an image. The higher order spectrum method is a typical signal processing method [18–21]; it has been developed rapidly over the past few years and offers outstanding performance in processing non-Gaussian, non-linear, non-minimum phase, and non-stationary signals, colored Gaussian noise, and blind signals [22]. High order statistics (HOS) have been proved to provide abundant diagnostic information [23–26]. Bi-spectrum is a subset of higher order spectrum and is defined in terms of the third-order cumulate. It retains phase information of the signal with a high resistance to noise [9, 27]. Therefore, the bi-spectrum contour map is employed to realize the transformation from vibration signals to images.

Owing to the development of the image automatic feature extraction technique in recent decades, the scale invariant feature transform (SIFT) method is recognized as the most appealing descriptor for practical uses and for matching features with good robustness and high accuracy [28–30]. However, some obvious shortcomings associate with SIFT, such as high resource consumption, high time complexity, and large computational time requirements [31]. To address these disadvantages, researchers have implemented various improvements to SIFT [32–36]. In 2008, Bay et al. proposed the modified SIFT method, speeded up robust features (SURF) [36], which provides faster and better performance in the feature point detection and description scheme. SURF is combined with integral images and Haar wavelets with the characteristics of rotation and scale invariance. As a novel feature extraction algorithm, SURF not only brings an obvious advantage in the area of computational speed, but also performs excellent in repeatability, distinctiveness and robustness equal or close to previous methods. Rotating machinery is susceptible to interference of working conditions and noise, the invariance of SURF can extract more stability fault features. Therefore, SURF is employed to extract fault features from the transformed bi-spectrum contour map.

The SURF algorithm generates a 64-dimensional feature vector for each detected feature point. Considering the high dimensionality may result in feature redundancy and a waste of resources for subsequent calculation, it is necessary to reduce the dimensionality of the SURF feature vector. As an emerging dimensionality reduction technique, t-distributed stochastic neighbor embedding (t-SNE) can maintain the consistency of neighborhood probability distribution between high-dimensional and low-dimensional space, thus avoiding information loss as much as possible [37]. Therefore, t-SNE is used to reduce the dimensionality of the SURF feature vectors. Finally, a classical neural network, probabilistic neural network (PNN) is introduced for identification of fault modes.

Two typical types of rotating machinery, axial piston hydraulic pump and self-priming centrifugal pump, are selected to verify the proposed fault diagnosis method based on image processing. The experimental results demonstrate that the proposed method reaches a high accuracy.

## Methodology

**Fig 1:**
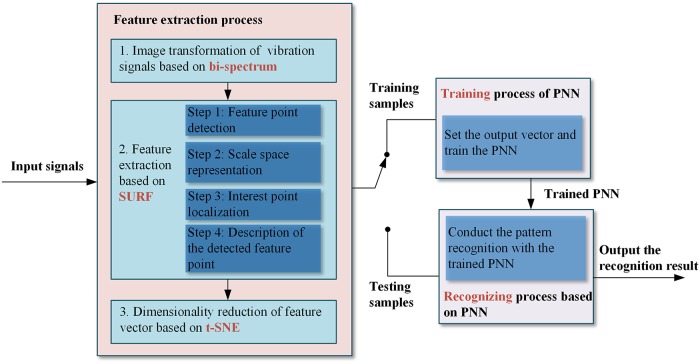
The proposed fault diagnosis scheme based on image processing.

The proposed method contains four major step: (1) Image transformation of vibration signal based on bi-spectrum. (2) Feature extraction based on SURF (3) Dimension reduction based on t-SNE (4) Fault diagnosis based on PNN. Fig 1 shows the proposed fault diagnosis scheme.

### Bi-spectrum counter map generation

A vibration signal of rotating machinery is a complex signal with non-liner, non-stationary signal with working noise, bi-spectrum is an effective tool to process such signal. Therefore, bi-spectrum technology is applied to transform vibration signal into image (bi-spectrum contour map), which prepare for the application of image processing technology.

Higher-order spectrum are defined by higher-order cumulant. So does bi-spectrum, which is defined by third-order cumulant. For a random signal *x*(*t*), the third-order cumulant can be expressed as:

$$C_{3x}\left(\tau_{1},\;\tau_{2}\right)=E\left\{x\left(t\right)x\left(t+\tau_{1}\right)x\left(t+\tau_{2}\right)\right\}$$ (1)

By assuming that the third-order cumulant *C*_3*x*_(*τ*_1_, *τ*_2_) is convergent, the *third-order* cumulant spectrum is defined as the 2-dimensional Fourier transform of the *third-order* cumulant:

$$B_{x}\left(w_{1},w_{2}\right)=\overset{\infty }{\underset{\tau_{1}=-\infty }{\sum }}\sum_{\tau_{2}=-\infty }C_{3x}\left(\tau_{1},\tau_{2}\right)e^{-j\left(w_{1}\tau_{1}+w_{2}\tau_{2}\right)}$$ (2)

The bi-spectrum of a signal should be estimated under a certain “optimum” standard. In practice, the bi-spectrum can be estimated only according to limited observation data. The estimated method of the bi-spectrum method is the same as the power spectrum estimation method, including the parametric model and non-parametric model. Compared with the indirect method, less computation is required by the direct method [38, 39]. Therefore, the direct estimation method is adopted in this paper. The flow of the algorithm is described as follows:

1. By assuming that the observation data are of finite length, the sampling frequency is *f*_*s*_. In the bi-spectrum domain, the number of the points are *ω*_1_ and *ω*_2_, and thus the frequency sampling interval is Δ_0_ = *f*_*s*_*/N*_0_. Divide *x*(*t*) into *k* segments, with each segment containing *M* points; (i.e., *N = KM*), then subtract the mean of each sample.
2. The discrete flourier transform (DFT) is applied for the *j*th data segment; that is:
  $$X^{j}\left(\lambda \right)=\frac{1}{M}\overset{M}{\underset{i=1}{\sum }}x^{j}\left(i\right)\text{exp}\left(-j\frac{2\pi }{M}i\lambda \right)$$ (3)
3. According to the coefficient of DFT, calculate the bi-spectrum estimation of each segment:
  $$B_{x}^{j}\left(\lambda_{1},\lambda_{2}\right)=\frac{1}{\Delta_{0}^{2}}\overset{L}{\underset{k_{1}=-L_{1}}{\sum }}\overset{L}{\underset{k_{2}=-L_{1}}{\sum }}X^{j}\left(\lambda_{1}+k_{1}\right)X^{j}\left(\lambda_{2}+k_{2}\right)X^{j}\left(\lambda_{1}+k_{1}+\lambda_{2}+k_{2}\right)$$ (4)
4. According to the result of the bi-spectrum estimation $B_{xx}^{j}\left(\lambda_{1},\lambda_{2}\right)$ of each segment, calculate the mean of $B_{xx}^{j}\left(\lambda_{1},\lambda_{2}\right)$ and then obtain the bi-spectrum estimate of the observation data *x*(*i*):
  $$B_{x}\left(\omega_{1},\omega_{2}\right)=\frac{1}{K}\overset{k}{\underset{j=1}{\sum }}B_{x}^{j}\left(\omega_{1},\omega_{2}\right)$$ (5)
  Where *ω*_1_ = [2*πf*_*s*_/*N*_0_]*λ*_1_, *ω*_2_ = [2*πf*_*s*_/*N*_0_]*λ*_2_.

### Feature extraction based on SURF

After the completion of the image conversion, the next step is to use the technology of image processing to extract the fault information contained in the bi-spectrum contour map. As a classical method in the field of image processing, SURF process the advantages of high precision and high speed. Its application is has been found in the field of visual tracking, underwater motion estimation, and other related fields for its novelty. Herein, SURF is employed to extract and describe feature points of the bi-spectrum contour map. The basic procedure is as follows:

#### Feature point detection

Feature point detection in the SURF algorithm is based on Hessian matrices; the feature point is located according to the local maximum of the Hessian matrix. Consider a certain point *(x*, *y)* in row *x*, column *y* and image *I*. The Hessian matrix *H*(*x*, *σ*) at x with scale *σ* Gaussian filter is defined as:

$$H\left(x,\sigma \right)=\left[\begin{array}{ll} L_{xx}\left(x,\sigma \right) & L_{xy}\left(x,\sigma \right)\\ L_{xx}\left(x,\sigma \right) & L_{yy}\left(x,\sigma \right) \end{array}\right]$$ (6)

where *L*_*xx*_(*x*, *σ*), *L*_*xy*_(*x*, *σ*), *L*_*yy*_(*x*, *σ*) are the second-order partial derivative and two-dimension convolution of point (*x*, *y*) of the image *I*, respectively.

**Fig 2:**
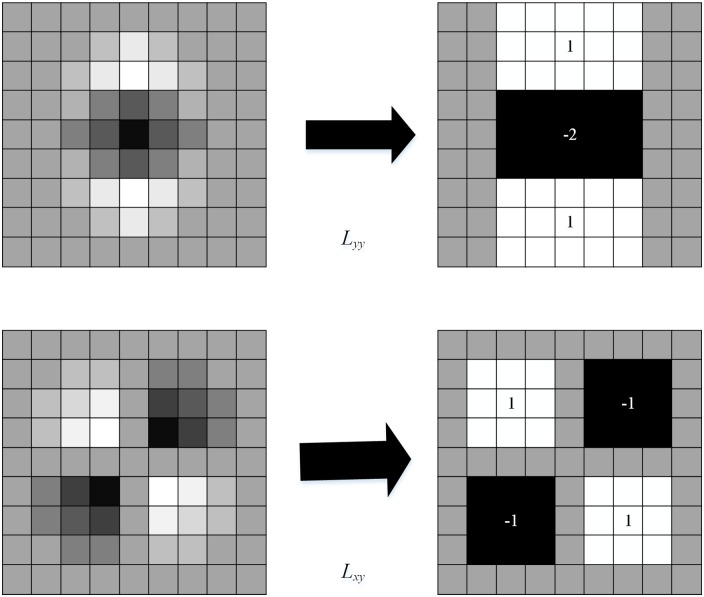
Approximated second order derivatives with box filters.

The Gaussian function is applicable in scale-space analysis, but in practical application process es, it needs to be discretized and cropped, as shown in the left column of Fig 2. This results in a loss in repeatability during the process of image rotation around odd multiples of *π*/4. However, it is inevitable that it be discretized and cropped. Given Lowe’s success in approximating LoG with DoG, Bay improved the approximation for the second-order Gaussian derivative even further with box filters, as shown in right column of Fig 2. There is a clear benefit for processing integral images with a convolution template after approximation. The template consists of a simple rectangle whose computation is independent of the size of the template. Hence, the computational efficiency is greatly accelerated.

In [36], experimental data indicates that the approximation does not degrade the performance of the convolution template. The convolution template in Fig 2 are approximations of a Gaussian function with *σ* = 1.2, which is the lowest scale for calculating the blob response maps. We denote the approximation template and convolution by *D*_*xx*_, *D*_*yy*_ and *D*_*xy*_, which are used instead of *L*_*xx*_(*x*, *σ*), *L*_*xy*_(*x*, *σ*), and *L*_*yy*_(*x*, *σ*) to obtain the approximate Hessian matrix H. That is:

$$\left|H\right|=D_{xy}\left(X\right)D_{xy}\left(X\right)-{\left[0.9D_{xy}\left(X\right)\right]}^{2}$$ (7)

where *w* is set as 0.9. According to (1), we calculate and record the response of each point to obtain the response map in scale *σ*.

#### Scale space representation

To search the feature points in the image with reducing-enlarging relation, the detection operator should have the ability to search the feature point in the same physical location at different scale. Scale space is usually donated by an image pyramid. The images are repeatedly smoothed with a Gaussian filter and then sub-sampled for sake of achieving a higher level of the pyramid. Lowe subtracts these pyramid layers to obtain the DoG (Difference of Gaussians) images where edges and blobs can be found.

**Fig 3:**
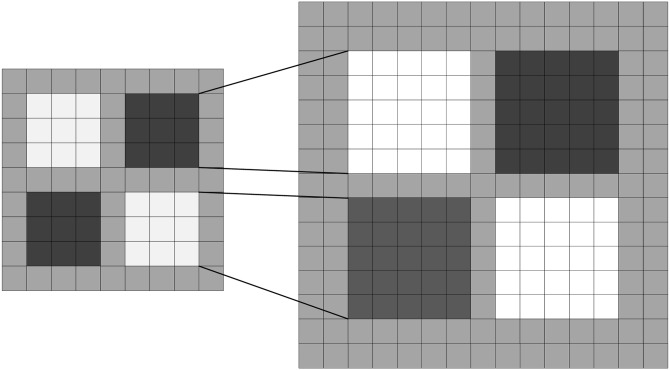
Filters *D*_*xy*_ for two successive scale levels (9×9 and 15×15).

In contrast to this method, SURF processes the original image with box filters of different size. Owning to the application of an integral image, the computation speed between box filters of different size are the same. In the left column of Fig 3, two 9*9 approximate templates are considered to be an initial scale template (approximating Gaussian derivatives with *σ* = 1.2). S refers to the scale of approximate template, where s = *σ* = 1.2. The initial layer is noted as the convolution between approximate templates of the initial scale of the image. The following layers are obtained by the convolution between gradually increasing s and the initial image. To guarantee the existence of cardinality and its central pixel, adjacent templates always differ by an even number.

Each octave consists of 4 templates. In the first octave, adjacent templates differ by 6 pixels; in the second octave, adjacent templates differ by 8 pixel. In the third octave, adjacent templates differ by 24 pixels, and so on. If the size of a template is N*N, the scale of the template is S = 1.2*9/N. We calculate the response at each point and record the response; the response map can be obtained at a different scale. Thus, a 3-dimension scale space can be constructed.

#### Interest point localization

We seek the extremum of scale image in (*x*, *y*, *σ*) according to the Fast-Hessian matrix. First, non-maxima suppression is performed at the extreme points of the 3×3×3 circular neighborhood. Only extreme points greater or less than 26 compared to the neighborhood value of the scale adjacent up and down and the scale itself can be selected as feature points. To locate candidate feature points with sub-pixel resolution, we interpolate between scale space and image space to obtain a stable feature point and its scale value in its location.

#### Description of the detected feature point

**Fig 4:**
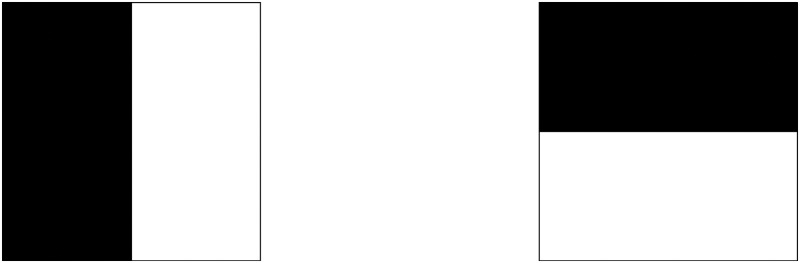
Haar wavelet filters.

To enable the descriptor to exhibit rotation invariance, the direction of feature point should be obtained first. Construct a wavelet response whose center is a feature point and whose radius is 6 s (s is the scale of the feature point). Process the image with Haar wavelet of size 4 s; the wavelet response in the direction of the x-axis and y-axis can be obtained. Haar wavelet templates are shown in Fig 4. The template on the left is used to calculate the response in the x-axis direction, whereas the one on the right is used to calculate the response in the y-axis direction. The black parts are denoted by -1, and the white parts are denoted by +1.

**Fig 5:**
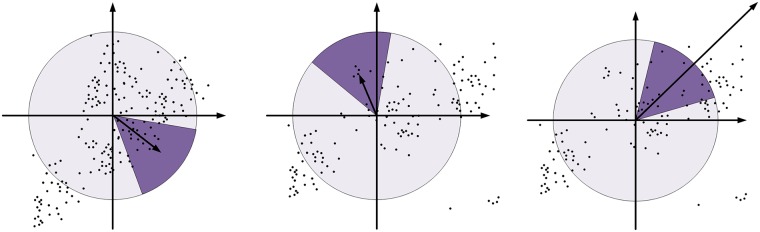
Determining the main direction of the feature point.

The neighborhood of the wavelet response is in the directions of the x-axis and y-axis; assign different Gaussian weight coefficients to these responses. A local direction vector is formed by summing the Harr wavelet in the direction of the x-axis and y-axis within 60°. Traverse the entire circle, then select the direction of the longest vector as the main direction of this feature point. The main direction determination of the feature point is illustrated in Fig 5.

#### Construct SURF feature vector

Construct a window region whose center is a feature point and whose side length is 20*σ*. Next, divide the window region into 4×4 sub-regions. A 5×5 sampling point is obtained from the sub-region. Seek the wavelet response in the directions of the x-axis and y-axis of each sampling point, donated as *dx* and *dy*, respectively. A Gaussian filter is applied to *dx* and *dy* in each sub-region. The filter center is taken as the feature point, and a four-dimensional eigenvector (Σ*dx*, Σ*dy*, Σ|*dx*|, Σ|*dy*|) is formed by summing *dx*, *dy*, |*dx*|, |*dy*| in each sub-region. The four-dimensional eigenvector makes up 4 dimensions of the descriptor. Each descriptor consists of 4 dimensions. Thus, 4×4×4 = 64 dimensions are obtained and constitute the SURF descriptor.

### Dimensionality reduction based on t-SNE

The feature points extracted by SURF are described in terms of a 64-dimensional descriptor. The high dimensionality will result in feature redundancy and a waste of resources for subsequent calculation. Therefore, it is necessary to reduce the dimensionality of the SURF feature vector. Manifold learning methods are widely used dimensionality reduction methods, which can be divided into two types, linear methods and nonlinear methods. Linear manifold learning methods include principal component analysis (PCA), multidimensional scaling (MDS), etc.; nonlinear manifold learning methods include Isometric Feature Mapping (Isomap), locally-linear bedding (LLE), etc[40].

Stochastic neighbor embedding (SNE) is one of the best performed nonlinear manifold learning algorithm whose core idea is to maintain the consistency of neighborhood probability distribution between high-dimensional and low-dimensional space. SNE transfers traditional Euclidean distance-based similarity measurement to conditional probability-based similarity measurement: in high dimensional observation space, the Gaussian distribution is adopted to simulate the similarity relationship between observation samples. Similarity between *x*_*i*_ and *x*_*j*_ *p*_j|i_ is denoted as follows:

$$p_{\text{j}|\text{i}}=\frac{\text{exp}\left(-\|x_{j}-x_{i}{\|}^{2}/2\sigma_{i}{}^{2}\right)}{\sum_{k\neq i}\text{exp}-\|x_{j}-x_{i}{\|}^{2}/2\sigma_{i}{}^{2}}$$ (8)

Where *σ*_i_ is the bandwidth of the Gaussian kernel function in the observation sample *x*_*i*_. *p*_j|i_ is the probability of *x*_*j*_ chosen by *x*_*i*_ as its neighbor sample. The parameter *p*_j|i_ obeys a Gaussian distribution in which the variance is $\sigma_{i}^{2}$ and the mean value is *x*_*i*_. The probability of *x*_*i*_ and *x*_*j*_ being adjacent to each other is

$$p_{ij}=\frac{p_{j|i}+p_{i|j}}{2n}$$ (9)

In low-dimensional space, SNE continues to adopt the Gaussian distribution to measure the similarity between low-distinction samples. However, two obvious shortcomings exists with SNE: (1) the objective function is too complex to optimize, and the gradient is not as concise as desired; (2) the so-called “crowding problem”; that is, when the data are far apart from each other in high-dimensional space, they must be gathered in the process of mapping to low-dimensional space. In response, t-distributed stochastic neighbor embedding (t-SNE) is introduced to alleviate these problems[37].

To solve the first problem, the characteristic of symmetry is adopted to simplify the objective function and optimize the gradient form, which is referred to as symmetric SNE. According to probability theory, the SNE objective function minimizes the sum of distances of the conditional probability distribution *p*_j|i_ (high-dimension) and *p*_i|j_ (low-dimension) for corresponding points. It equals the two following joint probability distributions of P (high-dimension) and Q (low-dimension):

$$C=KL\left(P\|Q\right)=\underset{i}{\sum }\underset{j}{\sum }p_{ij}\text{log}\frac{p_{ij}}{q_{ij}}$$ (10)

After adopting a joint probability distribution instead of a conditional probability distribution, the formula is more concise and understandable.

As for the second question, the t-distribution function is introduce to alleviate the “crowding problem”. That is, the t-distribution function is used to measure the similarity of points in low-dimensional space. The joint probability distribution function is as follows:

$$q_{ij}=\frac{{\left(1+\|y_{i}-y_{j}{\|}_{2}^{2}\right)}^{-1}}{\underset{k\neq 1}{\sum }{\left(1+\|y_{i}-y_{j}{\|}_{2}^{2}\right)}^{-1}}$$ (11)

Here, the t-distribution function (DOF is 1) is applied because of its special advantageous characteristic: ${\left(1\text{+}\|y_{i}-y_{j}{\|}_{2}^{2}\right)}^{-1}$ is the reciprocal of the distance of points far from each other in low-dimensional space to ∥*y*_*i*_−*y*_*k*_∥^2^. That means that in low-dimensional space, the presentation of the joint probability distribution is insensitive to the distance of points. In addition, in theory, the t-distribution function offers the same performance as the Gaussian function because the t-distribution function can be express as the infinity Gaussian function. Thus, the gradient of t-SNE is:

$$\frac{\delta C}{\delta y_{i}}=4\underset{j}{\sum }\left(p_{ij}-q_{ij}\right)\left(y_{i}-y_{j}\right){\left(1+\|y_{i}-y_{j}{\|}_{2}^{2}\right)}^{-1}$$ (12)

In conclusion, t-SNE focuses on:

1. The characteristic of non-similarity is associated with points far from each other. The characteristic of similarity is associated with points close to each other. The t-distribution function is introduced to exert an “exclusion” to process the non-similar points;
2. The application of the t-distribution function make it easier to optimize.

The t-SNE approach applies a probability density distribution function to measure the similarity and distribution characteristics. Then, t-SNE accomplishes property preservation by minimizing the K-L distance probability density in high-dimensional and low-dimensional space. The t-distribution function is employed to measure the similarity of points in low-dimensional space, which simplifies the gradient form and improves computational speed. Most important is to better process the non-similarity points and thus alleviate the “crowding problem”.

In most research reports, t-SNE exhibits better performance than Sammon mapping, isomap and locally linear embedding.

### Fault diagnosis based on PNN

PNN is a feedforward neural network developed from radial basis function; its theoretical basis is the Bayes minimum risk rule (Bayes decision theory). As one of the radial basis function networks, it can be used for pattern recognition[41].

Fault diagnosis based on PNN is a generally acceptable decision-making method in probability statistics. By assuming faults mode A and B, for fault feature sample X to be recognized, according to Bayes minimum risk rule, if *H*_*A*_*L*_*A*_*F*_*A*_(*X*) > *H*_*B*_*L*_*B*_*F*_*B*_(*X*), *X* ∈ *A*; if *H*_*A*_*L*_*A*_*F*_*A*_(*X*) < *H*_*B*_*L*_*B*_*F*_*B*_(*X*), *X* ∈ *B*. Here, *H*_*A*_ and *H*_*B*_ denote the prior probability of fault mode A and B. *L*_*A*_ is the cost factor of classifying feature X sample (belonging to A) into mode B falsely. *L*_*B*_ is the cost factor of classifying feature sample X (belonging to B) into mode A falsely. *F*_*A*_ and *F*_*B*_ are the density functions of fault mode A, B. Generally, they can only be obtained by the statistic of the fault feature sample, which cannot be obtained precisely. The density function can be estimated as follows:

$$F_{A}\left(X\right)=\frac{1}{{\left(2\pi \right)}^{p/2}\delta^{p}}\frac{1}{m_{t}}\sum \text{exp}\left[-\frac{{\left(X-X_{Ai}\right)}^{T}\left(X-X_{Ai}\right)}{2\delta^{2}}\right]$$ (13)

where *X*_*Ai*_ is the *i* th training vector of fault mode A; *m*_*t*_ is the total number of training samples of fault mode A; and *δ* is a smoothing parameter whose value determines the width of the mitriform curve centered on samples.

**Fig 6:**
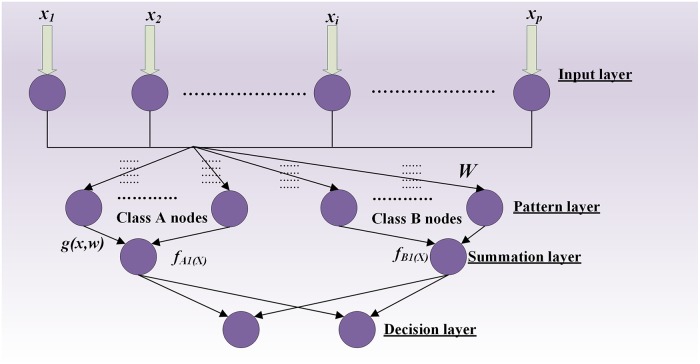
Basic structure of PNN.

PNN is a feedforward neural network with a parallel 4-year structure: input layer, pattern layer, summation layer and output layer, as shown in Fig 6. The input layer passes input samples to each node of the pattern layer; the pattern layer weighted summation input vector is passed by input nodes, which then pass it to the summation layer after calculation by the non-linear operator. The purpose of the summation layer is to sum up the input from the pattern layer and obtain the estimated probability densities. The classification result selected by the output layer is the maximum of the output of the summation layer in terms of the corresponding classification results.

## Experiment Results

In order to verify the effectiveness of the proposed method, two typical rotating machinery, self-priming centrifugal pump and axial piston hydraulic pump are selected for case verification.

### Case 1. Fault diagnosis for self-priming centrifugal pump based on SURF and t-SNE

#### (1) Data preparation

**Fig 7:**
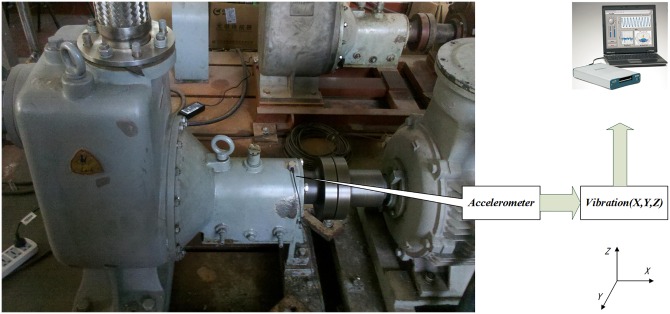
Self-priming centrifugal pump data acquisition system.

The data of self-priming centrifugal pump are collected on a self-priming centrifugal pump data acquisition system, as shown in Fig 7. The acceleration sensor is installed above the motor housing, and the sensor is fixed on a specific pedestal.

According to the requirement of fault diagnosis for centrifugal pump, a data acquisition experimental scheme is created for the fault insertion test. The test covers primarily fault modes. The experiment items are listed in Table 1.

**Table 1:** Experiment items of centrifugal pumps fault injection.

Test object	Failure test	Normal test
**rolling bearings**	bearing inner race wearing test	Bearing normal operation test
	bearing outer race wearing test	
	bearing rollers wearing test	
**Impeller**	impeller wearing test	

In the experiment, the rotation speed is 2,900/min. An acceleration sensor is employed when sampling. The sample frequency is 10239Hz.

Vibration data are collected under normal conditions and fault conditions, including bearing roller wearing, inner race wearing, and outer race wearing fault conditions, as well as impeller wearing fault condition. The sampling time is 2s for each set, and one set is collected every 5 seconds.

#### (2) Feature extraction based on SURF and t-SNE

**Fig 8:**
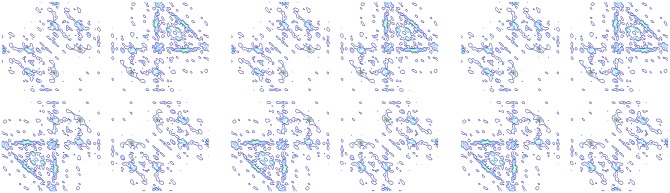
Bi-spectrum counter maps under bearing roller wearing fault condition.

**Fig 9:**
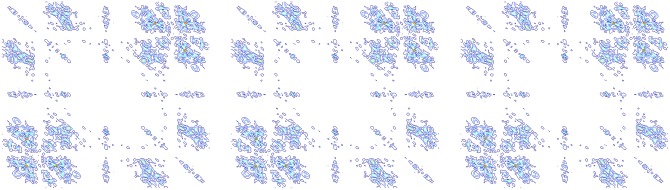
Bi-spectrum counter maps under bearing inner race wearing fault condition.

**Fig 10:**
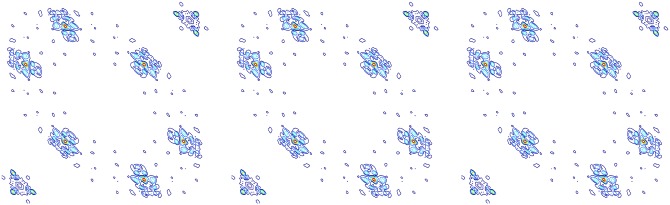
Bi-spectrum counter maps under bearing outer race wearing fault condition.

**Fig 11:**
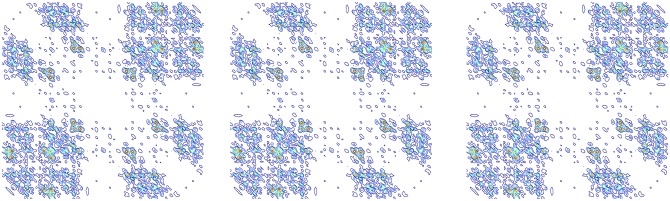
Bi-spectrum counter maps under normal condition.

**Fig 12:**
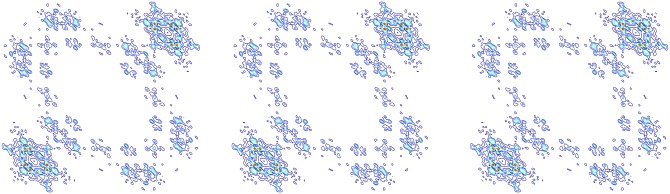
Bi-spectrum counter maps under impeller wearing fault condition.

Figs 8–12 shows the bi-spectrum counter maps of bearing roller wearing, inner race wearing, outer race wearing, and normal conditions, and centrifugal pump impeller wearing fault condition, respectively. For each condition, three groups of data sets are selected to generate bi-spectrum counter maps for comparison.

**Fig 13:**
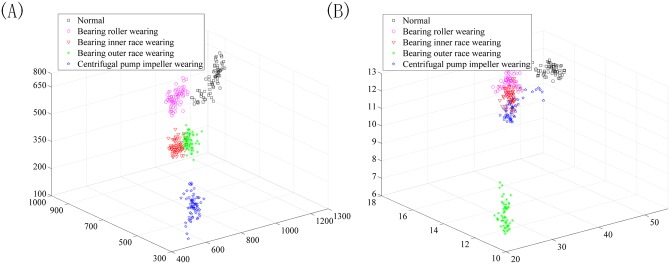
The first three features extracted using t-SNE (a) and without using t-SNE (b).

In the experiment, the SURF descriptor extracts the feature point of the bi-spectrum with 64-dimension form. By using t-SNE, the origin features of the datasets are reduced automatically to 20 dimensions. Fig 13 shows the distribution of the first three features extracted using t-SNE and without using t-SNE, respectively. From the first three features, we can conclude that the feature information is more concentrated and exhibits a strong ability for separability after dimension reduction by t-SNE, which provides a desirable input for classification.

#### (3) Fault diagnosis result based on PNN

The input eigenvector of PNN is extracted by SURF and t-SNE. To display the result visually, the classification result is contrasted with the actual result. The accuracy rate is defined as the odds ratio of the correct results and the total results.

Four set cross validation is adopted to verify the accuracy of the proposed method. For each fault mode, 60 sets of data are collected. The length of the data is 1024. Divide the 60 sets into 4 groups; each group is selected as training data in turn, whereas the others are selected as test data. The composition of the data is shown as Table 2.

**Table 2:** The data composition of the self-priming centrifugal pump for cross validation.

	bearing roller wearing	bearing inner race wearing	normal	centrifugal pump impeller wearing	the bearing outer race wearing	total amount
**number of data points**	60	60	60	60	60	300
**amount of training data**	15	15	15	15	15	75
**amount of test data**	45	45	45	45	45	225

**Fig 14:**
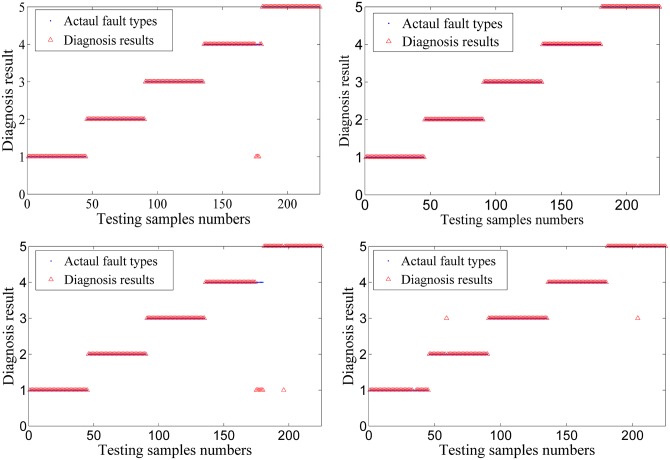
Results of 4 groups of cross validation.

The fault diagnosis of PNN is shown as follows. Fig 14 show the results of 4 set cross validation. The red circle is the actual fault category, and the blue triangle is the fault diagnosis result. Annotations 1~5 in vertical axis represent the bearing roller wearing, the bearing inner race wearing, the normal condition, the centrifugal pump impeller wearing and the bearing outer race wearing. Table 3 presents a summary of the cross-validation results.

**Table 3:** The error rate of 4 groups of cross-validation.

Cross-validation No.		bearing roller wearing	bearing inner race wearing	normal	centrifugal pump impeller wearing	bearing outer race wearing	Mean value/total
**1**	samples in test set	45	45	45	45	45	225
	error samples	0	0	0	2	0	2
	accuracy	1	1	1	0.9556	1	0.9911
**2**	samples in test set	45	45	45	45	45	135
	error samples	0	0	0	0	0	0
	accuracy	0	0	0	0	0	1
**3**	samples in test set	45	45	45	45	45	225
	error samples	0	0	0	6	1	9
	accuracy	1	1	1	0.8667	0.9778	0.9600
**4**	samples in test set	45	45	45	45	45	225
	error samples	2	1	0	0	1	4
	accuracy	0.9556	0.9778	1	1	0.9778	0.9822

From the diagnosis of PNN we can conclude that all of the accuracy rates exceed 96%. The cross- validation results of the first, second, third, and fourth sets are 99.11%, 100.00%, 96%, and 98.22%, respectively. Average classification accuracy is as high as 98.33%, which verifies the effectiveness of the proposed method.

### Case 2. Fault diagnosis for axial piston hydraulic pump based on SURF and t-SNE

#### (1) Data preparation

**Fig 15:**
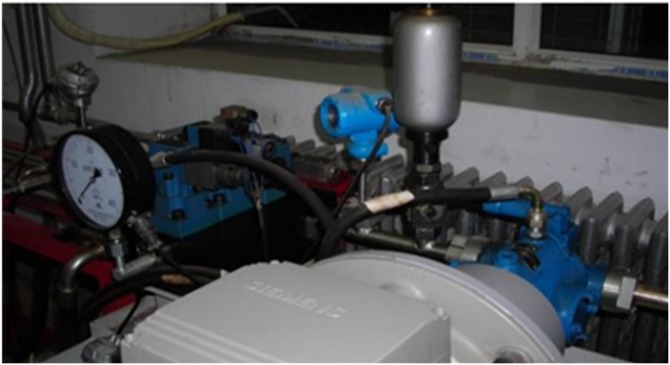
Axial piston hydraulic pump system.

The testing equipment for the axial piston hydraulic pump is shown in Fig 15. In the experiment, the rotation speed is set as 5280r/min and the corresponding spindle frequency is 88Hz. An accelerograph is installed at the end face of the pump. The sample frequency is 1k Hz. The data collected contains fault modes of normal, piston shoes and swashplate wearing, and valve plate wearing. A data set of 500 points for each team is selected for analysis.

#### (2) Feature extraction based on SURF and t-SNE

**Fig 16:**
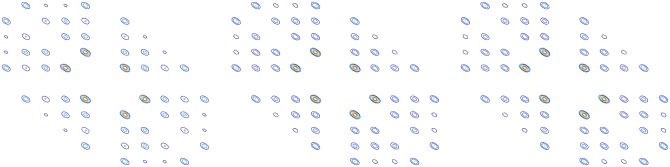
Bi-spectrum counter map of normal.

**Fig 17:**
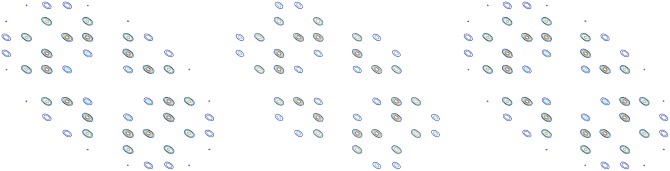
Bi-spectrum counter map of valve plate wearing.

**Fig 18:**
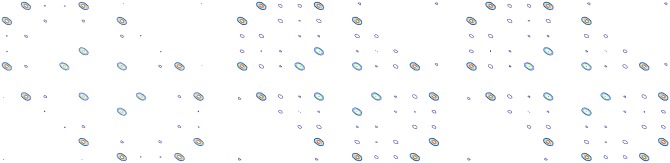
Bi-spectrum counter map of piston shoes and swashplate wearing.

Bi-spectrum estimation using the direct method for an axial piston hydraulic pump system is conducted. We segment each sample into M records of 512 points each. The spectrum contour map of normal, piston shoes and swashplate wearing, and valve plate wearing are shown in Figs 16–18, respectively. The bi-spectrum of three data sets are selected for comparison.

From the spectrum contour it can be observed that the same types of fault mode are similar; although there are obvious differences between different fault modes, there are invariant features between the same fault modes. The differences between different fault modes can be used to distinguish fault types.

**Fig 19:**
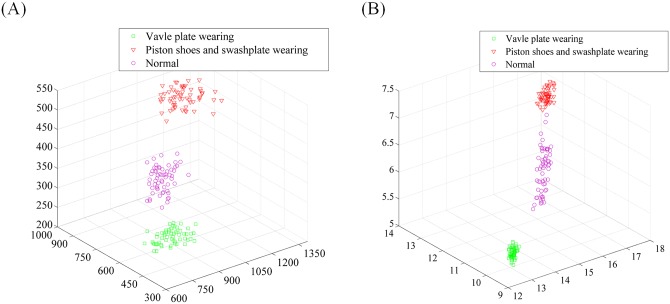
The first three features extracted using t-SNE (a) and without using t-SNE (b).

In the experiment, the SURF descriptor converts the bi-spectrum into a 64-dimension vector. Using t-SNE, the origin features of the datasets are reduced automatically to 20 dimensions. Fig 19 shows the distribution of the first three features extracted using t-SNE and without using t-SNE. From the first three features, we can conclude that the feature information is more concentrated and exhibits a strong ability of separability after dimension reduction by t-SNE, which provides a desirable input for classification.

#### (3) Fault diagnosis result based on PNN

The input eigenvector of PNN is extracted by SURF and t-SNE. To display the result visually, the classification result is contrasted with the actual result. The accuracy rate is defined as the odds ratio of the correct results and the total results.

Four set cross validation is adopted to verify the accuracy of the proposed method. For each fault mode, 60 sets of data are collected. The data set length is 500. We divide the 60 sets into 4 groups; each group is selected as training data in turn, whereas the others are selected as test data. The composition of the data is shown as Table 4.

**Table 4:** The data composition of axial piston hydraulic pump for cross validation.

	normal	piston shoes and swashplate wearing	valve plate wearing	Total
**number of data points**	60	60	60	180
**amount of training data**	15	15	15	45
**amount of testing data**	45	45	45	135

**Fig 20:**
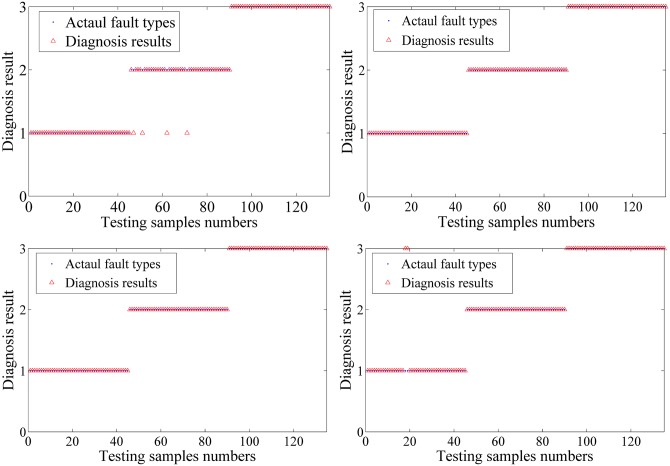
Result of 4 set cross validation.

The fault diagnosis of PNN is shown as follows. Fig 20 show the results of 4 sets cross validation. The red circle is the actual fault category, and the blue triangle is the fault diagnosis result. The numbers 1~3 in the vertical axis represent normal, piston shoes and swashplate wearing and valve plate wearing. Table 5 presents a summary of the cross validation results.

**Table 5:** The error rate of 4 groups of cross-validation.

No. of cross validation		Normal	piston shoes and swashplate wearing	valve plate wearing	mean value/total
**1**	samples in test set	45	45	45	135
	error samples	0	4	0	4
	accuracy	100%	91.11%	100%	97.04%
**2**	samples in test set	45	45	45	135
	error samples	0	0	0	0
	accuracy	100%	100%	100%	100%
**3**	samples in test set	45	45	45	135
	error samples	0	0	0	0
	accuracy	100%	100%	100%	100%
**4**	samples in test set	45	45	45	135
	error samples	2	0	0	2
	accuracy	95.56%	100%	100%	97.78%

From the results diagnosed by PNN, we can conclude that the accuracy rate of all four set cross validation tests has exceeded to 97%. The cross- validation results of the first, second, third, and fourth sets are 97.04%, 100.00%, 100.00%, and 97.78%, respectively. Average classification accuracy is as high as 98.71%, which verifies the effectiveness of the proposed method.

## Conclusions

In this paper, we present a novel rotating machinery diagnosis method based on image recognition that contains four major steps: First, the bi-spectrum is employed to transform the initial vibration signal into an image (bi-spectrum counter map). Next, SURF is first introduced to extract feature points of the image automatically. To reduce the dimension while describing the feature points accurately as much as possible, the manifold dimension reduction method t-SNE is used to map the high-dimensional features to low-dimensional space. Based on the feature vectors extracted by SURF and t-SNE, PNN is applied to enable fault mode recognition.

The proposed image-recognition-based fault diagnostic method for rotating machine first introduces the image interest point extraction method to provide a fault diagnosis and achieve feature extraction of the bi-spectrum automatically. Thus, the method avoids the limitations of relying on a diagnostician for feature extraction. Favorable results for two types of typical rotating machinery demonstrate that our method can improve robustness and generalization ability while maintaining accuracy of classification. Our subsequent work will be focused as follows:

1. Apply this method to more types of machinery.
2. Improve computing speed while maintaining diagnostic accuracy.
3. Conduct further research about the fault diagnosis technologies with fluctuated working conditions.

## Supporting Information

S1 File The raw data of self-priming centrifugal pump. (RAR)

S2 File The raw data of hydraulic pump. (RAR)
